# Respiratory syncytial virus hospitalization in children in northern Spain

**DOI:** 10.1371/journal.pone.0206474

**Published:** 2018-11-15

**Authors:** Natividad Viguria, Iván Martínez-Baz, Laura Moreno-Galarraga, Luis Sierrasesúmaga, Blanca Salcedo, Jesús Castilla

**Affiliations:** 1 Department of Pediatrics, Complejo Hospitalario de Navarra, Pamplona, Spain; 2 Instituto de Investigación Sanitaria de Navarra, IdiSNA, Pamplona, Spain; 3 Instituto de Salud Pública de Navarra, Pamplona, Spain; 4 CIBER Epidemiología y Salud Pública, Pamplona, Spain; 5 Clínica Universidad de Navarra, Pamplona, Spain; 6 Servicio de Gestión, Información y Evaluación, Complejo Hospitalario de Navarra, Pamplona, Spain; University of Cape Town, SOUTH AFRICA

## Abstract

**Objectives:**

Understanding respiratory syncytial virus (RSV) morbidity may help to plan health care and future vaccine recommendations. We aim to describe the characteristics and temporal distribution of children diagnosed with RSV admitted in a Spanish hospital.

**Methods:**

Descriptive study for which the hospital discharges of children < 5 years of age with RSV infection were analyzed. The information was extracted from the hospital discharge database of a reference pediatric hospital in northern Spain for the 2010–2011 to 2014–2015 RSV seasons.

**Results:**

Six hundred and forty-seven hospitalizations of children with RSV infection were analyzed, 94% of which occurred between the second week of November and the last week of March. Most children (72%) were under one year of age and 95% were previously healthy infants. Infants born from October to December had the highest risk of hospitalization in the first year of life. The median length of hospital stay of children with and without comorbidities was six and three days, respectively. 6.5% of the hospitalized cases were admitted to the pediatric intensive care unit; this percentage was higher among children < 2 months (adjusted odds ratio 4.15; 95% confidence interval: 1.37–12.61) or with comorbidities (adjusted odds ratio 4.15; 95% confidence interval: 1.53–11.28). The case lethality was 0.3%.

**Conclusions:**

The risk of hospitalizations for RSV is high during the first year of life and increases among infants born in the fall. Being under two months of age and presenting comorbidities are the main risk factors associated to pediatric intensive care unit admission.

## Introduction

Respiratory syncytial virus (RSV) lower respiratory tract infection is a very common hospital admission cause worldwide [1, 2]. Its primary clinical diagnosis is acute bronchiolitis (AB), which leads to complications such as acute respiratory failure and apneas in children younger than six weeks of age [3]. Up to 22% of the affected children may require admission to the pediatric intensive care unit (PICU) [4]. In preterm infants, bronchopulmonary dysplasia, congenital heart disease, Down’s syndrome, neuromuscular diseases and other chronic diseases, are contributors for worse clinical outcomes and higher risk of mortality [5, 6]. In these cases, immune prophylaxis with monthly injections of palivizumab is recommended during the RSV season [7]. Early initiation of immune prophylaxis increases the costs, as more than five doses are necessary to protect a child for the whole season, while a late initiation leave them unprotected during the beginning of the season [8]. There are geographical variations at the start and end of the outbreak and understand them can help optimize immune prophylaxis strategies. The correlation between microbiological detections of RSV and hospital admissions to determine when to begin immune prophylaxis has been described elsewhere [9].

Furthermore, being under six months of age at the start of the RSV season has been associated to a higher risk of hospital admissions due to RSV infection [10, 11]. In Spain, a greater risk of admission due to RSV has been observed in infants born during the Spring/Winter season [12]. However, this also depends on the geographical variations at the start and end of the RSV epidemic with respect to the climate [13, 14].

The aim was to describe the frequency, temporal distribution, and epidemiological characteristics in children under five years of age admitted in a reference hospital with a diagnosis of RSV between 2010 and 2015; furthermore, we will analyze the characteristics associated to the admission in the PICU.

## Material and methods

### Study design and sources of information

A descriptive observational study was conducted in a tertiary pediatric hospital in the North of Spain, covering a population of around 32,056 five-year-old minors by January 2016 [15]. The Regional Health Service provides health care, free at point of service, to 97% of the Navarra population. Since 1997, all hospitalizations are included in an electronic hospital discharge database in which the diagnosis at discharge is codified following the International Classification of Diseases, Ninth revision, Clinical Modification (ICD-9-CM).

All data were fully anonymized before accessing them. The Navarra Committee for Clinical Research approved the study protocol (160629 Pyto 2016/42).

Hospitalizations of children < 5 years of age between 2010 and 2015 were obtained from the hospital discharge database with specific diagnosis of RSV, including the following ICD-9-CM codes among primary or secondary conditions: acute bronchiolitis (code 466.11), pneumonia (code 480.1), and RSV-associated infections (code 079.6) [16].

Furthermore, we also used the database from the hospital-based Microbiology Laboratory. Confirmation of RSV was done by using a rapid immunochromatography test for RSV detection (BinaxNOW RSV Card, Alere Scarborough Inc., Maine, USA) and/or a molecular reverse transcriptase assay,—real-time PCR for detection of RSV and flu (RealCycler FLURSV, Progenie Molecular, Valencia, Spain). Either one or both assays are systematically performed on all children admitted due to respiratory infection.

The following data were collected for each episode: sex, date of birth, hospital admission and discharge dates, and place were the patient was admitted and direct or intermediate transfers to PICU, length (days) of hospital stay, primary diagnosis and all secondary diagnosis at discharge.

Regarding comorbidities the following ICD-9-CM diagnostic codes were considered: bronchopulmonary dysplasia (770.7x), congenital respiratory malformations (748.xx), congenital heart and vascular anomalies (745.0x-745.9x; 746.0x-746.9x; 747.0x-747.9x), cystic fibrosis (277.0x), neuromuscular disorders (330.xx; 335.xx; 343.xx; 356.xx; 358.1; 359.0x-23; 359.9; 775.2), Down’s syndrome without congenital heart disease (758.0x), immunodeficiency (279.xx), other congenital and metabolic disorders (740.xx; 741.xx; 742.xx; 754.2; 756.1x; 756.6; 758.1–758.9; 759.3; 759.7x-759.9x; 271.0; 272.7; 277.5; 277.81; 277.82; 277.86), and history of low birth weight (< 2,500 grams) including code V21.3x [17].

The following ICD-9-CM codes were indicative of severity: acute respiratory failure (518.81), hypoxemia (799.02), acute respiratory distress (518.82), and apnea (786.03) [18]. Patients who required mechanical ventilation were also considered as in severe condition (ICD-9-CM procedure codes 93.90, 96.01, 96.02, 96.03, 96.04, 96.05, 96.70, 96.71, 96.72).

To define the onset and the end of the RSV season, the definition of the Pediatric Investigators Collaborative Network on Infections in Canada (PICNIC) was used. This definition establishes the beginning of the outbreak as the Monday before two consecutive seven-day periods during which there were more than two hospital admissions diagnosed with RSV and the end of the outbreak the Monday before two consecutive seven-day periods during which there was one or no admission due to RSV [8, 19]. Should an increase of RSV cases occur after the initially established end date, the RSV outbreak was considered to continue until the end criteria is met.

### Statistical analysis

Quantitative variables were expressed as means and standard deviations (SD) or medians and interquartile ranges (IQR), and were compared using the Mann-Whitney U-test. Categorical variables were presented as percentages and comparisons made with the Pearson’s chi-square test. Differences between children depending on their month of birth, presence of comorbidities, and admission to PICU were assessed by calculating the odds ratio (OR) with 95% confidence interval (CI). Logistic regression adjusted for age, sex, comorbidity, diagnosis at discharge, and season was applied to evaluate the factors associated to PICU admission. A statistical software package (SPSS Version 20) was used for the analysis of the data. P values below 0.05 were considered statistically significant.

## Results

### Variability of admissions during RSV seasons

There were 647 hospitalizations due to RSV between the 2010–2011 and 2014–2015 seasons; the admission rate of patients < 5 years of age was 3.8/1,000 children-season (95% CI: 3–4). The hospitalization rate varied depending on the age. In infants with less than two months the hospitalization rate was 33.3 admissions/1,000 (95% CI: 29–38), in those younger than one year this rate was 14.1 admissions/1,000 (95% CI: 13–15), and in children aged between 1–4 years, 1.32 admissions/1,000 children-season (95% CI: 1–2) Table 1.

**Table 1:** Characteristics of children under 5 years of age admitted to the hospital with a diagnosis of respiratory syncytial virus grouped per periods.

	2010–2011	2011–2012	2012–2013	2013–2014	2014–2015	Total
	n (%)	n (%)	n (%)	n (%)	n (%)	n (%)
**Total**	119 (100)	184 (100)	142 (100)	133 (100)	69 (100)	647 (100)
**Age groups**						
< 2 months	29 (24)	45 (25)	38 (27)	51 (38)	19 (27)	182 (28)
2–11 months	54 (45)	89 (48)	65 (46)	43 (32)	30 (43)	281 (43)
1 year	22 (18)	34 (18)	25 (18)	21 (16)	13 (19)	115 (18)
2–4 years	14 (12)	16 (9)	14 (10)	18 (13)	7 (10)	69 (11)
**Rate of hospitalization/1,000 children-season (95% CI)**						
<2 months	25.8(17–35)	40.3(29–52)	34.7(24–46)	51,1(37–65)	18,4(10–27)	33.3(29–38)
<1 year (0–11 months)	12.3(10–15)	20.0(17–23)	15.6(13–19)	15.7(13–19)	7.9(6–10)	14.1(13–15)
1–4 years	1.27 (1–2)	1.76 (1–2)	1.38 (1–2)	1.41 (1–2)	0.75 (0–1)	1.32 (1–2)
**Sex male**	70 (59)	105 (57)	83 (58)	66 (50)	44 (64)	368 (57)
**RT-PCR positive for RSV**	34 (29)	98 (53)	38 (27)	121 (91)	58 (84)	349 (54)
**Length of hospital stay (days), median (interquartile range)**	4 (3–6)	4 (3–5)	3 (2–4)	3 (2–4)	3 (2–3)	3 (2–5)
**Mean length of hospital stay (days) (SD)**	5.3 (6.6)	6.4 (18.2)	3.5 (2.5)	4.2 (8.5)	3.1 (2.4)	4.8 (11.0)
Range	(1–56)	(1–183)	(1–15)	(1–72)	(1–14)	(1–183)
**Disease due to RSV as primary diagnosis**	97 (81)	150 (82)	110 (78)	115 (87)	64 (93)	536 (83)
**Diagnosis at discharge**						
Pneumonia due to RSV	18 (15)	22 (12)	10 (7)	12 (9)	1 (1)	63 (10)
Acute bronchiolitis due to RSV	91 (77)	141 (77)	117 (82)	108 (81)	64 (93)	521 (80)
Infection due to RSV	12 (8)	21 (11)	15 (11)	13 (10)	4 (6)	63 (10)
**Severity**	44 (37)	60 (33)	62 (44)	59 (44)	15 (22)	240 (37)
Acute respiratory failure	36 (30)	54 (29)	59 (42)	55 (41)	12 (17)	216 (33)
Apnea	2 (2)	1 (1)	1 (1)	3 (2)	0 (0)	7 (1)
**Mechanical ventilation**	1 (1)	8 (4)	1 (1)	4 (3)	1 (1)	15 (2)
**Comorbidity**	9 (8)	14 (8)	4 (3)	4 (3)	4 (6)	35 (5)
Low birth weight	5 (4)	4 (2)	0 (0)	1 (1)	2 (2)	12 (2)
Bronchopulmonary dysplasia	0 (0)	3 (2)	0 (0)	0 (0)	1 (1)	4 (1)
Congenital respiratory malformation	0 (0)	1 (1)	0 (0)	0 (0)	0 (0)	1 (0)
Congenital heart anomaly	1 (1)	3 (2)	0 (0)	0 (0)	1 (1)	5 (1)
Cystic fibrosis	0 (0)	0 (0)	0 (0)	0 (0)	0 (0)	0 (0)
Neuromuscular disorder	1 (1)	2 (1)	2 (1)	0 (0)	0 (0)	5 (1)
Down´s syndrome	2 (2)	1 (1)	0 (0)	0 (0)	1 (1)	4 (1)
Immunodeficiency	0 (0)	0 (0)	0 (0)	0 (0)	0 (0)	0 (0)
Other congenital and metabolic diseases	1 (1)	3 (2)	2 (1)	3 (2)	0 (0)	9 (1)

RT-PCR: reverse transcriptase—polymerase chain reaction, SD: standard deviation, RSV: respiratory syncytial virus.

Considering total admissions per year, there was variability between periods in the percentage of children < 5 years admitted due to RSV: 4.3% (119/2781) for the 2010–2011 period, 7.1% (184/2576) for 2011–2012, 6.5% (142/2255) for 2012–2013, 6.3% (133/2040) for 2013–2014, and 3.3% (69/2064) for 2014–2015.

The most outstanding change was the decrease of admissions due to RSV during the 2014–2015 period in comparison to the previous ones (*p* < 0.001).

**Fig 1:**
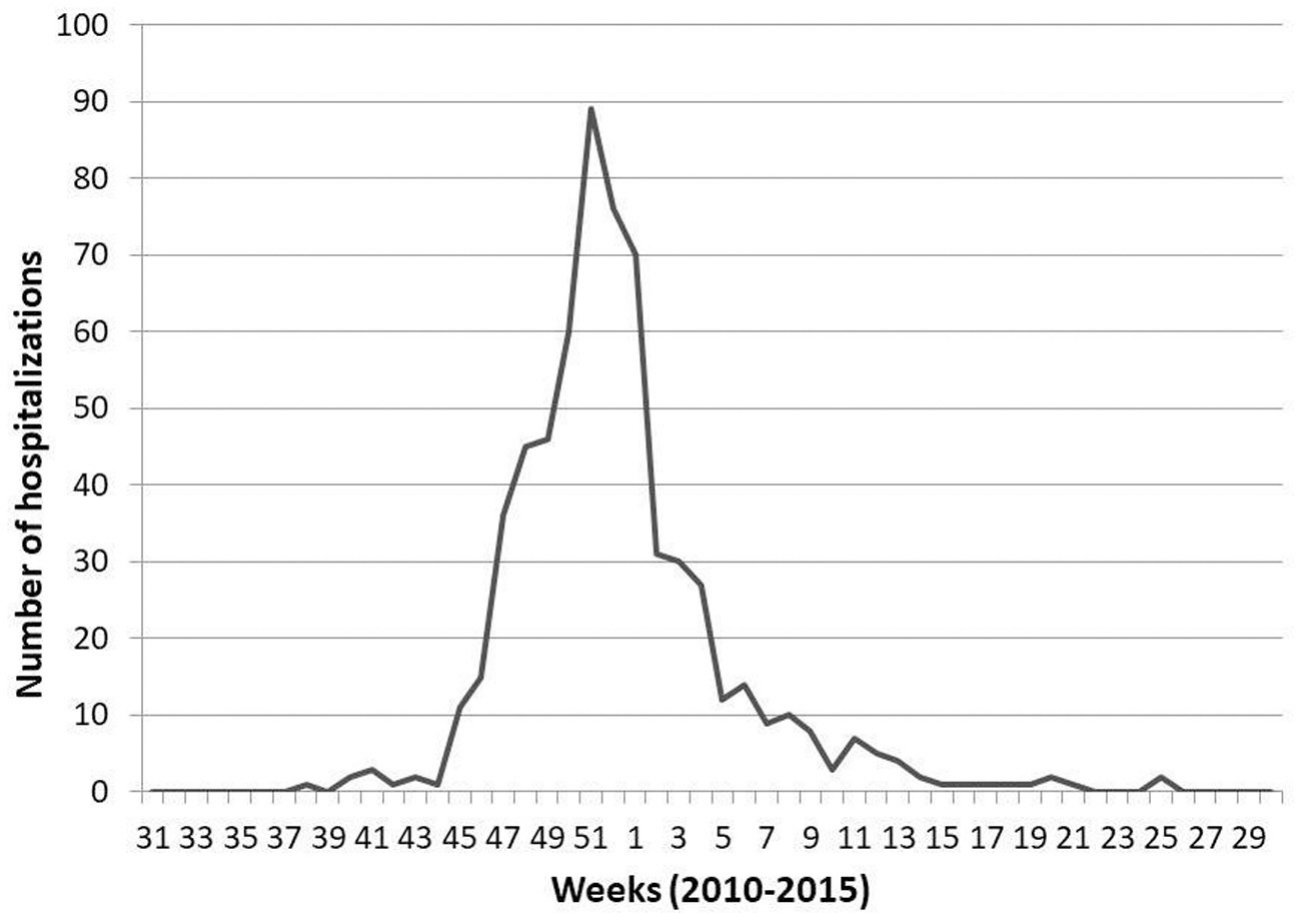
Weekly hospital admissions of children diagnosed with respiratory syncytial virus throughout the 2010–2011 to 2014–2015 seasons.

The admissions associated to a diagnosis of RSV showed strong seasonality. During the 2010–2015 period, there were not admissions in July; moreover, there were only sporadic admissions in June, August, and September of the same period. The beginning of the RSV outbreak occurred between weeks 45 to 48 (November) and the end between weeks 8 to 15 (February-April); the peak of the outbreak was between weeks 50 to 52 (December). RSV season duration was between 12 (2012–2013) and 21 weeks (2014–2015). Considering all the periods, 94% (608/647) of the admissions due to RSV occurred between week 45 (second week of November) and week 13 (last week of March); the highest average of admissions was on week 51 (penultimate of the year) (Fig 1).

### Month of birth as admission risk factor in children under one year of age

**Fig 2:**
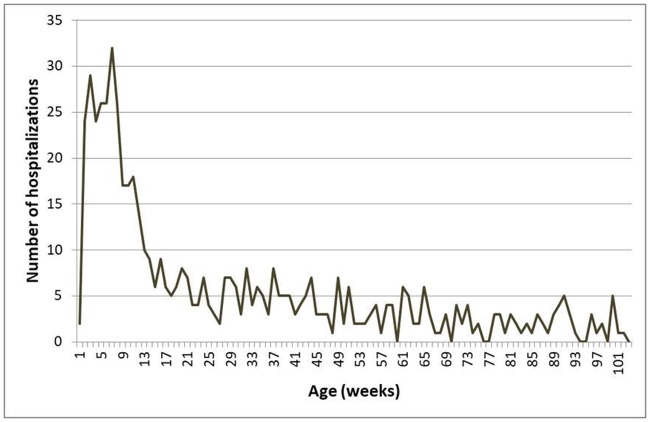
Hospital admissions of children diagnosed with respiratory syncytial virus per age (weeks) throughout the seasons 2010–2011 to 2014–2015.

Fifty-seven per cent of the admitted children in the study period were boys; 28% were younger than eight weeks and 72% under one year of age (Table 1). The number of admissions was high throughout the first year of life, with a maximum at week seven after birth (Fig 2).

**Fig 3:**
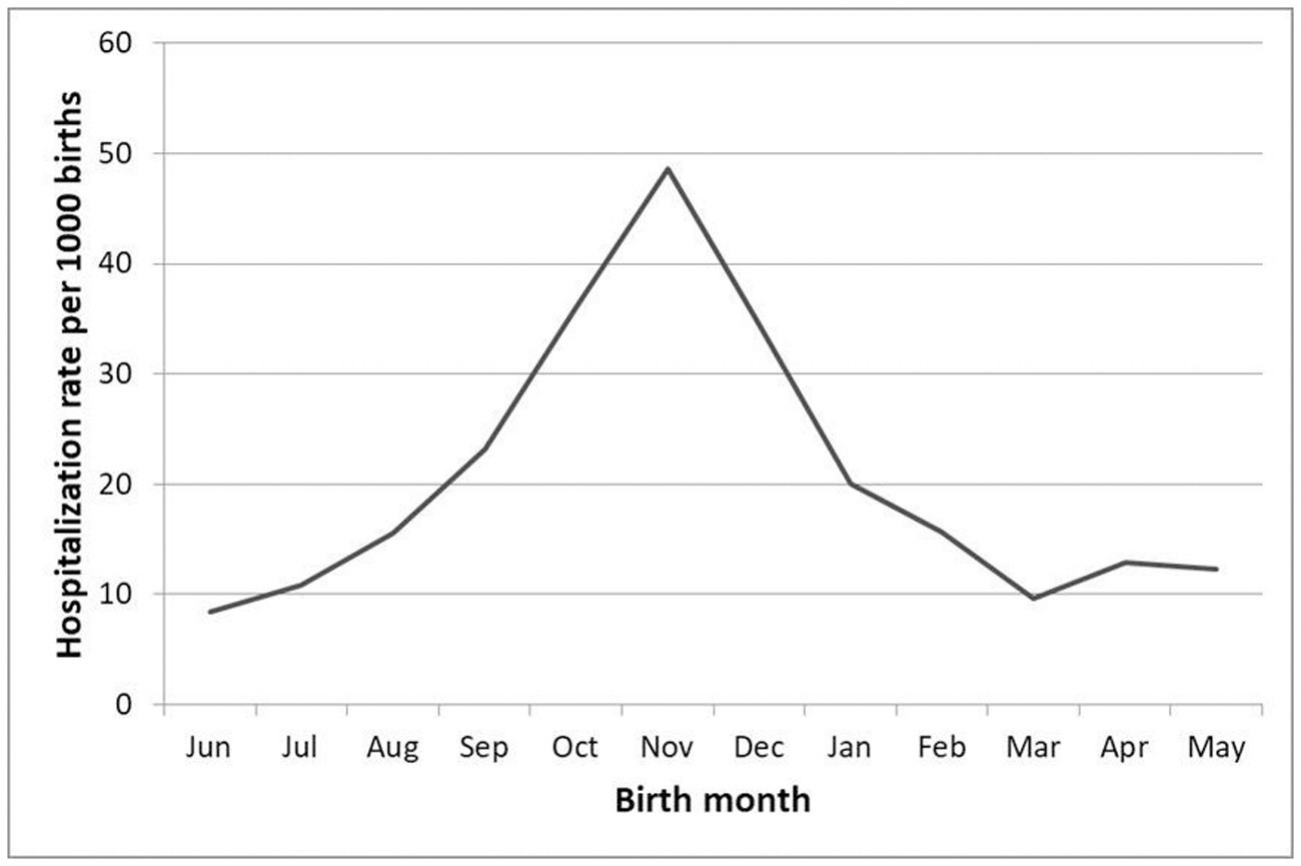
Average hospitalization rate of children under one year of age diagnosed with respiratory syncytial virus as per the month of birth throughout the 2010–2011 to 2014–2015 seasons.

Among study children, 2.1% were admitted due to RSV before reaching the age of one year (463 admissions/22,379 births). We assessed the risk of hospitalization associated to RSV during the first year taking into account the month of birth. The lowest risk was determined for children born in June (0.8%; 95% CI: 0.4–1.3; 16/1900) and the highest risk was distributed between those born in October (3.6%; 95% CI: 2.8–4.4; 71/1972), November (4.9%; 95% CI: 3.9–5.8; 88/1811), and December (3.5%; 95% CI: 2.6–4.3; 65/1884) (Fig 3). Compared with children born in June showed that those under one year of age born in October (OR 4.4; 95% CI: 2.5–7.6), November (OR 6.0; 95% CI: 3.5–10.2), and December (OR 4.2; 95% CI: 2.4–7.3) had a higher risk of being admitted due to RSV.

### Molecular diagnosis

The microbiological diagnosis of RSV was performed with an immunochromatographic rapid test in 46% of the cases and with RT-PCR in 54%; the percentage of molecular diagnosis increased from 29% for the 2010–2011 period to 84% for the 2014–2015 one (Table 1).

In 83% of the admissions, RSV infection was the primary diagnosis at discharge with acute bronchiolitis being the most frequent diagnosis (80%).

### Median of length of hospital stay by age and comorbidity

The median length of hospital stay varied throughout the study period from 4 days (IQR 3–6) in 2010–2011 to a median of 3 days (IQR 2–3) in 2014–2015. Five per cent of the admissions showed some comorbidity or risk condition, the most frequent being low birth weight (2%). Amongst the severity criteria, in 33% of the cases acute respiratory failure was observed and apneas in 1% (both complications were seen in three of the study children).

In comparison to infants < 2 months, the rate of admission to the PICU was lower in those aged between 2–11 months (12% vs 4%, *p* < 0.001) (Table 2). No differences by sex or length of stay were observed.

**Table 2:** Analysis of hospitalization episodes with a diagnosis of respiratory syncytial virus in children under one year of age.

	Total*	< 2 months	2–11 months	p**
	n (%)	n (%)	n (%)	
**Total**	463 (100)	182 (100)	281 (100)	
**Male**	273 (59)	106 (58)	167 (59)	0.799
**Admission to the PICU**	32 (7)	22 (12)	10 (4)	<0.001
**Length of hospital stay (days), median (interquartile range)**	3 (2–5)	3 (2–5)	3 (2–4)	0.314
**Length of hospital stay (days), mean (SD)**	4.8 (11.1)	4.4 (5.2)	5.1 (15.0)	0.588
**Period**				0.021
2010–2011	83 (18)	29 (16)	54 (19)	
2011–2012	134 (29)	45 (25)	89 (32)	
2012–2013	103 (22)	38 (21)	65 (23)	
2013–2014	94 (20)	51 (28)	43 (15)	
2014–2015	49 (11)	19 (10)	30 (11)	

*Total number of children under one year of age

**Pearson’s chi-square test for comparing children under two years of age against those between 2 to 11 years

PICU: pediatric intensive care unit

Regarding comorbidities, children with one or more showed a longer median hospital stay (6 vs 3 days, *p* = 0.048), greater severity (54% vs 36%, *p* = 0.030), and were admitted to the PICU more frequently (17% vs 6%, *p* = 0.009). In 84% of the children without comorbidities, RSV was the primary diagnosis in comparison to 66% to children with comorbidities (*p* = 0.006) (Table 3).

**Table 3:** Comparison between children under five years of age hospitalized with a diagnosis of respiratory syncytial virus, with or without comorbidities.

	Total	With comorbidities	Without comorbidities	p*
	n (%)	n (%)	n (%)	
**Total**	647 (100)	35 (100)	612 (100)	
**Age group**				0.253
< 2 months	182 (28)	5 (14)	177 (29)	
2–11 months	281 (43)	17 (49)	264 (43)	
1 year	115 (18)	9 (26)	106 (17)	
2–4 years	69 (11)	4 (11)	65 (11)	
**Males**	368 (57)	16 (46)	352 (58)	0.170
**RT-PCR positive for RSV**	349 (54)	29 (83)	320 (52)	< 0.001
**Admission to PICU**	42 (7)	6 (17)	36 (6)	0.009
**Diagnosis at discharge**				0.055
Pneumonia due to RSV	63 (10)	5 (14)	58 (10)	
Acute bronchiolitis due to RSV	521 (81)	23 (66)	498 (81)	
Infection due to RSV	63 (10)	7 (20)	56 (9)	
**RSV as the primary diagnosis**	536 (83)	23 (66)	513 (84)	0.006
Severity**	240 (37)	19 (54)	221 (36)	0.030
**Hospital stay (days), median (interquartile range)**	3 (2–5)	6 (3–10)	3 (2–5)	0.048
**Length of hospital stay (days), mean (SD)**	4.8 (11.0)	18.4 (40.6)	4.0 (4.9)	< 0.001
**Period**				0.185
2010–2011	119 (18)	9 (26)	110 (18)	
2011–2012	184 (28)	14 (40)	170 (28)	
2012–2013	142 (22)	4 (11)	138 (23)	
2013–2014	133 (21)	4 (11)	129 (21)	
2014–2015	69 (11)	4 (11)	65 (10)	

*Pearson’s chi-square test

**Severity considered as a positive diagnosis at discharge of apnea, acute respiratory failure, hypoxemia, respiratory failure, or mechanical ventilation

RT-PCR: reverse transcriptase—polymerase chain reaction; PICU: pediatric intensive care unit; RSV: respiratory syncytial virus; SD: standard deviation

### Factors associated to PICU admission

Of all children discharged due to RSV younger than five years, 6.5% required direct admission or intermediate transfer to the PICU. In comparison to the children that did not need to go the PICU, in those who were admitted, the percentage of infants < 2 months was higher (52% vs 26%, *p* = 0.003), they had more comorbidities (14% vs 5%, p = 0.009), greater severity (69% vs 35%, *p* < 0.001), and showed a higher median of hospital stay (9 days, IQR 6–12.25 vs 3 days IQR 2–4, *p* < 0.001) (Table 4).

**Table 4:** Comparison of the characteristics of hospitalization episodes in children under five years of age with a diagnosis of respiratory syncytial virus, as per their admission to the PICU.

	Total	Admissions to the PICU	Not admitted in the PICU	p*
	n (%)	n (%)	n (%)	
**Total**	647 (100)	42 (100)	605 (100)	
**Age groups**				0.003
< 2 months	182 (28)	22 (52)	160 (26)	
2–11 months	281 (43)	10 (24)	271 (45)	
1 year	115 (18)	5 (12)	110 (18)	
2–4 years	69 (11)	5 (12)	64 (11)	
**Males**	368 (57)	24 (57)	344 (57)	0.971
**RT-PCR positive for RSV**	349 (54)	32 (76)	317 (52)	0.003
**Comorbidities**	35 (5)	6 (14)	29 (5)	0.009
**RSV as the primary diagnosis**	536 (83)	27 (64)	509 (84)	0.001
**Diagnosis at discharge**				0.107
Pneumonia due to RSV	63 (10)	4 (10)	59 (10)	
Acute bronchiolitis due to RSV	521 (80)	30 (71)	491 (81)	
Infection due to RSV	63 (10)	8 (19)	55 (9)	
Severity**	240 (37)	29 (69)	211 (35)	<0.001
**Length of hospital stay (days), median (interquartile range)**	3 (2–5)	9 (6–12.25)	3 (2–4)	<0.001
**Length of hospital stay (days), mean (SD)**	4.8 (11.0)	16.8 (30.2)	3.9 (7.5)	<0.001
**Period**				0.870
2010–2011	119 (18)	6 (14)	113 (19)	
2011–2012	184 (28)	14 (33)	170 (28)	
2012–2013	142 (22)	8 (19)	134 (22)	
2013–2014	133 (21)	10 (24)	123 (20)	
2014–2015	69 (11)	4 (9)	65 (11)	

* Pearson’s chi-square test

**Severity considered as a positive diagnosis at discharge of apnea, acute respiratory failure, hypoxemia, respiratory failure, or mechanical ventilation

PICU: pediatric intensive care unit; RT-PCR: reverse transcriptase—polymerase chain reaction; RSV: respiratory syncytial virus; SD: standard deviation

The multivariate analysis showed that having less than two months (OR = 4.15; CI 95%: 1.37–12.61) and the presence of comorbidity (OR = 4.15; CI 95%: 1.53–11.28) was associated to a greater risk of being admitted to the PICU. Throughout the various study periods, there were no significant changes in the risk of being admitted in the PICU (Table 5).

**Table 5:** Factors associated to pediatric intensive care unit admission among children diagnosed with respiratory syncytial virus.

	Crude OR (CI 95%)	*p*	Adjusted OR (CI 95%)	*p*
**Age groups**				
< 2 months	3.03 (1.11–8.23)	0.030	4.15 (1.37–12.61)	0.012
2–11 months	0.81 (0.27–2.43)	0.709	0.95 (0.29–3.07)	0.931
1 year	1		1	
2–4 years	1.72 (0.48–6.17)	0.406	1.76 (0.48–6.46)	0.397
**Sex**				
Female	1		1	
Male	1.01 (0.54–1.90)	0.971	1.09 (0.56–2.10)	0.804
**Comorbidity**				
No	1		1	
Yes	3.31 (1.29–8.49)	0.013	4.15 (1.53–11.28)	0.005
**Diagnostic at discharge**				
Pneumonia due to RSV	1.11 (0.38–3.26)	0.850	1.63 (0.47–5.63)	0.443
Acute bronchiolitis due to RSV	1		1	
Infection due to RSV	2.38 (1.04–5.45)	0.040	2.47 (1.03–5.94)	0.043
**Period**				
2010–2011	1		1	
2011–2012	1.55 (0.58–4.16)	0.383	1.63 (0.59–4.50)	0.345
2012–2013	1.12 (0.38–3.34)	0.833	1.24 (0.40–3.80)	0.710
2013–2014	1.53 (054–4.35)	0.424	1.46 (0.50–4.30)	0.491
2014–2015	1.16 (0.32–4.26)	0.824	1.36 (0.36–5.24)	0.652

OR: odds ratio obtained from logistic regression; CI: confidence interval; RSV: respiratory syncytial virus

### Lethality

During the study period, two of the children admitted due to RSV died. Both were under one year of age and had comorbidities: one of the boys had trisomy 18 and heart disease and died at 56 days of age; the second child presented severe brain damage and died at the age of seven months. The lethality among the children admitted for RSV was 0.3%.

## Discussion

The respiratory syncytial virus was a major cause of hospitalization in children younger than five years. Our rate of admission for these ages is similar to that described by Hall et al (3/1,000 children) [20] and Stein et al (4.37/1,000) [21]. However, large variations were seen depending on the age group, with the highest hospitalization rate being that of children under two months of age.

In our study, the admission rate greatly decreases in the 2014–2015 period, coinciding with changes in the clinical practice. The structure and facilities of the emergency department and the observation care unit underwent remodeling. Children remained in the observation unit up to 24 hours before hospital admission was considered; this reduced the number of admissions due to RSV or other causes [22].

Mean hospital length of stay due to RSV was similar to that reported in other Spanish studies [16, 23, 24]; however, the median length of stay was three days, which differs from some studies, such as one carried out in Denmark were the median for the cohort aged under five years of age was slightly shorter (1.96 days IQR 0.67–5.21) [25].

In this study, the risk of being admitted to the hospital due to RSV during their first year of life was higher for children born between October and December. The most probable explanation for this is that their first months of life was concurrent with the period of greatest circulation of the RSV; furthermore, RSV maternal antibodies undergo seasonal changes with lower titles at the beginning of the period when the mothers have not yet been exposed to the RSV [10, 26]. A study from the United Kingdom reported higher risk in children younger than one year born between September and November, coinciding with their RSV outbreak beginning slightly earlier (between weeks 41 to 43) [27]. In the USA, infants under one year born in December and January have a greater risk of being hospitalized [28]. In Spain, greater risk of being admitted due to RSV has been described in children born during the second half of the year [12]. These variations depict the different Autumn/Winter circulation patterns of the RSV in the various geographical areas of the Northern hemisphere.

We observed throughout the five study years an increased use of molecular techniques for microbiological diagnoses, as has been described by other authors. This increases our knowledge on the impact RSV, as compared to sites and periods were low-sensitivity rapid diagnostic tests were used [29].

A larger number of males were hospitalized. Some authors attribute this to a narrower diameter of the inferior distal airway tract [10] and others to the effect sexual hormones have on the immune response [30].

Here, we determined that being two months of age is a clear factor associated to hospital admission due to RSV, marking the turning point in the risk of severe forms of disease due to RSV. Research for preventing severe cases in children under two months currently focuses in the development of a vaccine for pregnant women [31, 32] or the administration of a long half-life monoclonal antibody with neutralizing activity against the RSV to the newborn [33].

Besides a longer length of hospital stay, as reported by other authors [16], children with comorbidities were admitted more frequently to the PICU, consequently making greater use of healthcare resources. This justifies the use of additional preventive measures, such as immune prophylaxis with palivizumab [7].

In our study 6.5% cases were attended in the PICU, a percentage slightly below the 10% described by Sirimi *et al*. in Greece. However, their study included children aged under 14 years for a period of 12 years (2002–2013), during which improvements have been made regarding intensive care therapeutic care [34]. The observed lethality among the children admitted for RSV is consistent with other studies, and deaths were related to severe comorbidities [35].

In the study region, with an oceanic climate (mean annual temperature of 12.3°C and mean annual rainfall of 1,042 mm [36]), 94% of the admissions occur between week 45 (second week of November) and week 13 (fourth week of March), with little interannual variability (3 weeks) at the start of the outbreak. When compared with other Mediterranean areas, some differences are observed. For example, in Malaga (mean annual temperature of 18.4°C and mean annual rainfall of 520 mm), the outbreak of acute bronchiolitis occurs from the fourth week of September and the third week of October (although it should be considered that the corresponding study included different agents that cause bronchiolitis) [37]. On the other hand, in Athens (mean annual temperature of 18.1°C and mean annual rainfall of 397 mm), the outbreak of RSV starts in December, reaches its peak in February, and ends in April [34].

This study presents the typical limitations of those based on hospital discharge databases, in which the care load due to RSV can be underestimated because of coding defects. However, the medical charts in our hospital are computerized, which increases reliability and the comparison with microbiological confirmations supports the validity of the diagnosis. Short hospital stays admissions (< 24 hours) in the emergency room department, are not registered in the hospital discharge databases.

The main strengths of our study are the inclusion of all admissions of RSV-affected children younger than five years in a tertiary reference hospital in the North of Spain, with a well-defined covered population, the availability of data for five years obtained with comparative criteria, and having the variables that allow establishing the start and the end of the outbreak, hospital stay, comorbidities, risk factors, admission to the PICU, and mortality. The hospital discharge database is an exhaustive information system in which all admissions are included; furthermore, regular audits are performed supporting the validity of the coding for the diagnoses and procedures. Having considered the primary and secondary hospitalization diagnoses prevents the underestimation of RSV cases in children with comorbidities, in which the RSV might not be registered as the primary diagnosis at discharge.

## Conclusions

RSV causes annual epidemics that are accompanied by a high number of hospital admissions in children during the first year of life. Despite improvements in healthcare, the number of admissions in PICU due to RSV over the years has remained the same, mostly being children < 2 months of age, who were previously healthy. Children with comorbidities are four times more at risk of being admitted to the PICU and are associated with highest mortality. Epidemiological surveillance of the RSV in each geographical area is required for adjusting health resources during the RSV epidemic, as well as to plan the immune prophylaxis.

The birth month is an important predictor of hospital admission in children under one year of age; in our environment the risk of admission increases further in children born between October and December.

## Supporting information

S1 Table Study database. Data_PONE_D_18_19911__1.
(XLS)
